# Reduced LDL-Cholesterol and Reduced Total Cholesterol as Potential Indicators of Early Cancer in Male Treatment-Naïve Cancer Patients With Pre-cachexia and Cachexia

**DOI:** 10.3389/fonc.2020.01262

**Published:** 2020-08-04

**Authors:** Hannes Zwickl, Klaus Hackner, Harald Köfeler, Eva-Christina Krzizek, Besnik Muqaku, Dietmar Pils, Hubert Scharnagl, Tora S. Solheim, Elisabeth Zwickl-Traxler, Martin Pecherstorfer

**Affiliations:** ^1^Department of Internal Medicine 2, Karl Landsteiner Private University of Health Sciences, University Hospital Krems, Krems an der Donau, Austria; ^2^Department of Pneumology, Karl Landsteiner Private University of Health Sciences, University Hospital Krems, Krems an der Donau, Austria; ^3^Core Facility Mass Spectrometry, Lipidomics Research Center Graz, Center for Medical Research (ZMF), Medical University Graz, Graz, Austria; ^4^Rudolfstiftung Hospital and Karl Landsteiner Institute of Obesity and Metabolic Diseases, First Medical Department, Vienna, Austria; ^5^Department of Analytical Chemistry, Faculty of Chemistry, University of Vienna, Vienna, Austria; ^6^Center for Medical Statistics, Informatics and Intelligent Systems, Medical University of Vienna, Vienna, Austria; ^7^Department of Surgery, Medical University of Vienna, Vienna, Austria; ^8^Clinical Institute of Medical and Chemical Laboratory Diagnostics, Medical University of Graz, Graz, Austria; ^9^Department of Clinical and Molecular Medicine, Faculty of Medicine and Health Sciences, NTNU - Norwegian University of Science and Technology, Trondheim, Norway; ^10^Cancer Clinic, St. Olavs Hospital, Trondheim University Hospital, Trondheim, Norway; ^11^Clinical Department of Internal Medicine II, Karl Landsteiner University of Health Sciences, Krems an der Donau, Austria

**Keywords:** cancer, cachexia, metabolism, cholesterol, metabolic syndrome

## Abstract

Cancer cachexia is characterized by the impairment of glucose and lipid homeostasis, the acceleration of processes promoting the mobilization of energy-rich compounds (e.g., insulin resistance, gluconeogenesis, and lipolysis) and the simultaneous activation of highly energy-demanding processes (e.g., systemic inflammation and activation of brown adipose tissue). We hypothesized that these processes might themselves change during cancer cachexia progression, such that plasma levels of glucose and lipids might be used to distinguish between the non-malignant state, pre-cachexia and cachexia. We performed an initial cross-sectional study including 60 treatment naïve cancer patients (38 with cancer cachexia and 22 with cancer pre-cachexia) and 61 patients without malignancy (21 with metabolic syndrome and 40 controls). Differences in lipids (total cholesterol, LDL and HDL cholesterol) and plasma fasting glucose were analyzed across various group configurations, with adjustments to age and antidiabetic or lipid-lowering drugs. Our study showed that levels of LDL cholesterol and total cholesterol might indicate cachexia stages irrespective of the presence of metabolic syndrome or lipid-lowering medication. High levels of plasma glucose were only seen in cachectic cancer patients on antidiabetics. These observations indicate that markers of metabolic dysregulation associated with cachexia progression might be exploited for early detection of malignancy.

## Introduction

Cancer cachexia is a major concern in clinical oncology, as it affects patients' response and tolerance to treatments, as well as quality of life and prognosis (1–4). Cancer cachexia is characterized by a negative energy balance due to an increased resting energy expenditure (5, 6) and anorexia (7–9), which results in the loss of body weight and muscle mass (10). Its pathophysiology is complex, involving systemic inflammation and central nervous mechanisms (7, 9, 10). The diagnostic criteria used to define the stages of cancer cachexia take these characteristics into account, though the extent of body weight loss is still the main criterion for differentiating between pre-cachexia^1^ and cachexia (2, 10–12).

Glucose and lipid homeostasis are impaired in cancer cachexia, due to the activation of processes promoting the mobilization of energy-rich compounds (9, 13), such as increased hepatic gluconeogenesis (14). Cancer cachexia is also associated with insulin resistance, which enhances both hepatic glycogenolysis (9, 15) and lipid mobilization from white adipose tissue (16). Lipolysis is increased, possibly by noradrenaline-mediated signaling involving the sympathetic nervous system (17–19). Moreover, cachexia-associated systemic inflammation and/or activation of brown adipose tissue are highly energy-demanding (17, 20), with the latter effectively removing energy-rich compounds from the systemic circulation found in normal physiology (21–23). Thus, the interplay of these metabolic processes might manifest in cachexia-related alterations of the plasma levels of energy-rich compounds (24). Indeed, plasma cholesterol has been shown to be reduced in patients with newly diagnosed solid tumors (24), as well as in lung cancer patients with different histological types (25, 26), while plasma triglycerides were either unaltered or decreased (26).

Browning of white adipose tissue also has been implicated in cancer cachexia (27). In normal physiology, browning is an adaptation to persistent cold stress, a response to an extended period of cold exposure (18). That browning and its thermogenic potential are triggered in cancer cachexia (18, 28) suggests that the demand for energy-rich compounds increases during cachexia progression (27). Thus, plasma levels of glucose and lipids might be distinguishing factors between the non-malignant state, pre-cachexia, and cachexia, indicating that the significance of these metabolic processes might change as cancer cachexia progresses.

Although its worldwide prevalence and geographic distribution are thought to vary widely on a region-by-region basis, metabolic syndrome (MeS) has been designated as a global epidemic, affecting an estimated 20% of adults in the western world (29, 30). The defining criteria of MeS are obesity, insulin resistance, increased plasma triglycerides (TG), decreased high-density lipoprotein cholesterol (HDL-C), and hypertension (30). Hence, MeS and cancer cachexia share insulin resistance as a central pathophysiological feature. Moreover, similar to MeS, cancer cachexia might affect plasma TG and HDL-C values. The control subjects in cancer cachexia studies typically are people without malignancy recruited from the normal population. Given the rates of MeS, this means that control groups likely consist of two distinct subgroups—with and without metabolic syndrome—with possible relevance for the proper interpretation of metabolic alterations due to cancer cachexia.

The overall aim of this study was to explore whether there were changes in lipid and glucose metabolism in pre-cachectic and cachectic cancer patients compared to a control group without malignancy. Some of these same metabolic changes may also characterize MeS. Therefore, we needed to find a way to tease out MeS-induced metabolic changes from cachexia-induced metabolic changes to determine whether any differences held up once we controlled for MeS in the control cohort and the intake of anti-diabetic or lipid-lowering medication in any group.

To elucidate differences between our control group of subjects without malignancy and pre-cachectic/cachectic cancer patients, we compared plasma levels of fasting glucose (Glc), TG, and HDL-C—three factors which are also defining criteria of MeS (30)—as well as levels of low-density lipoprotein cholesterol (LDL-C) and total cholesterol (Chol) across all three groups. In addition, by further stratifying the control group into two groups according to whether or not they exhibited the presence of metabolic syndrome, we investigated whether conclusions based on these three-group comparisons hold true in a four-group analysis. We also explored the potential influence of antidiabetic or lipid-lowering medications on parameters of interest in this study.

## Materials and Methods

### Patients and Study Design

Our research was conceived as a single-center, cross-sectional, explorative study. After approval of the study design by the Ethics Committee of the Federal State Lower Austria (GS1-EK-4/290-2014), all participants were recruited (and their written informed consent obtained) at the Krems University Hospital in Lower Austria between February 2016 and June 2018.

Subjects were eligible if they were male and over 40 years of age and did not suffer from chronic diseases of the cardiovascular system or insulin-dependent diabetes or exhibit an acute inflammatory disease state at the time of enrollment. The age restriction was put in place to (a) reduce potential age-related variability of the parameters to be investigated, and (b) facilitate the recruitment of cohorts of similar ages for our study.^2^ While no restriction was placed on types of cancer entities or stages of cancer, cancer patients had to be treatment naïve to be eligible. Due to sex differences in glucose and lipid metabolism (31), we restricted participation to male subjects, in order to limit the complexity of the dataset.

### Data Collection

Blood sampling was performed at Krems University Hospital between 8:00 and 10:00 a.m. after patients had fasted for at least 12 h. Cancer patients had their blood drawn after an overnight stay in the hospital before undergoing further clinically indicated examinations, while non-cancer patients were tested as walk-ins after fasting at home. The same day, participants demographic and anthropometric data (including waist circumference and blood pressure), as well as information on antidiabetic and lipid-lowering (hypolipidemic) medications were collected. In addition, cancer patients were asked to provide estimates of the date of onset of body weight loss and their pre-onset body weight. Date of onset was used to segment cancer patients into groups with pre-cachexia and cachexia. Data on cancer entity and stage were retrieved retroactively from the Krems University Hospital Oncology Information System (OIS) database.

Laboratory diagnostics were performed at the Krems University Hospital. Blood panel parameters included plasma levels of glucose^3^, triglycerides, total cholesterol, HDL and LDL cholesterol, C-reactive protein (CRP), albumin, and hemoglobin^4^. Plasma levels of LDL-C were analytically determined using the Friedewald equation (32).

### Statistical Considerations

For three-group analyses, study participants were assigned to one of two groups: non-malignant subjects or cancer patients, with the latter further subdivided into pre-cachectic or cachectic cancer patients according to the extent of their body weight loss (2, 10). Cancer patients were assigned to the cachectic group if their body weight loss was either ≥ 5% during the last 6 months or ≥ 2% coupled with a body mass index (BMI) of <20 kg/m^2^. Cancer patients with <5% body weight loss were assigned to the non-/pre-cachectic group. Data on additional criteria commonly used in cachexia scoring systems—C-reactive protein (CRP) ≥ 0.5 mg/dL, plasma albumin <3.2 g/dL, and hemoglobin <12 g/dL (1, 2)—also were collected to further characterize subjects, but in the absence of consensus on threshold values, were not used to distinguish between pre-cachectic and cachectic cancer patients.

The NM group was subdivided into a metabolic syndrome (MeS) and a control (Ctrl) group. Subjects were assigned to MeS if they had a waist circumference ≥ 94 cm, plus at least two of the following criteria: fasting plasma glucose ≥ 100 mg/dL, triglycerides ≥ 150 mg/dL, HDL-C <40 mg/dL, and blood pressure ≥ 130 mmHg (systolic) or ≥ 85 mmHg (diastolic) (33). Otherwise they were assigned to Ctrl. Subjects were not screened for the presence of metabolic syndrome prior to their enrollment, but were assigned to the respective groups a posteriori.

Plasma concentrations of glucose, triglycerides, HDL-C, LDL-C, and cholesterol were compared between (i) healthy persons (Ctrl), (ii) patients with metabolic syndrome (MeS), (iii) pre-cancer cachexia (pCC) and (iv) cancer cachexia (CC), with all four groups used in 4-group comparison and the first two (MeS and Ctrl) being combined into one group for 3-group comparisons (i.e., contrasts). Multiple regression analyses were performed, always including age and intake of analyte-relevant medication as putative confounders. Calculations were performed using the general linear hypotheses (glht) function of the R package multcomp^5^ 1.4-10 (PMID: 18481363). The significance of the individual group comparisons was estimated according to the above-defined contrasts according to Tukey's all-pair comparisons and corrected for multiple testing with the single-step method (adjusted *P*-values). Study participants' characteristics were analyzed via Kruskal-Wallis test and Dunn's multiple comparison test (adjusted *P*-values).

### Limitations

1. We enrolled only male subjects in this study for reasons explained in Section Patients and Study Design. Whether the results of this study hold true for female subjects requires further investigation.

2. Our study was cross-sectional across relatively small cohorts. The next step would be to study whether our findings hold true in a longitudinal study with larger cohorts over a longer period of time.

## Results

### Description of Study Population

**Figure 1:**
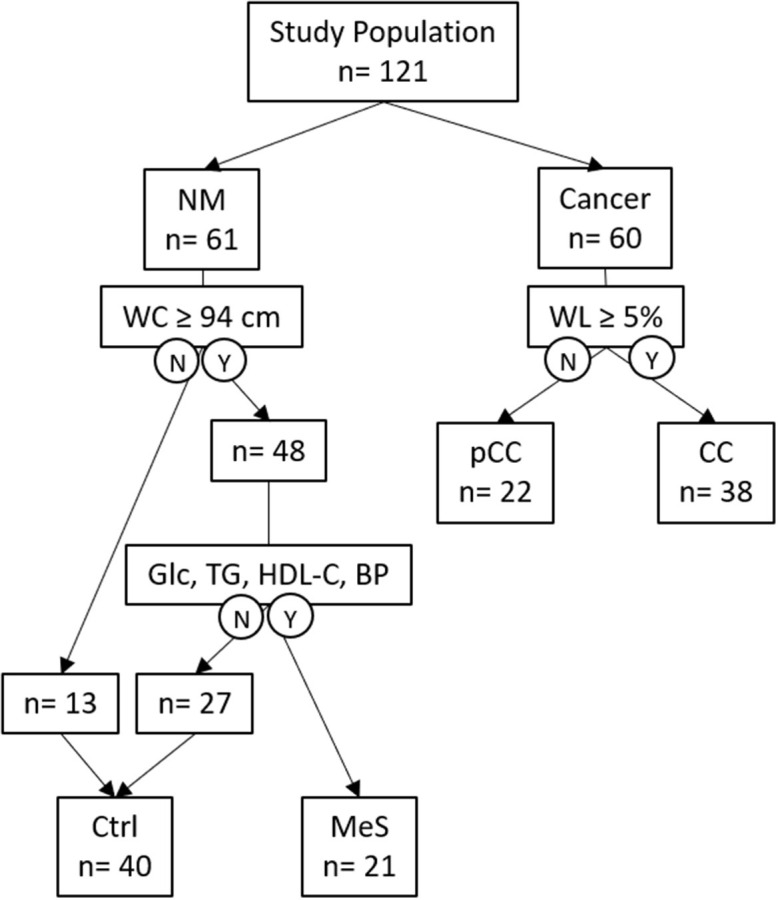
Overview of the study population outlining groups and features relevant to the study. NM, non-malignant; WL, weight loss; pCC; pre-cancer cachexia; CC, cancer cachexia; N, No; Y, Yes; WC, waist circumference; Glc, fasting plasma glucose; TG, triglycerides; HDL-C, high-density lipoprotein cholesterol; BP, blood pressure (systolic or diastolic); Ctrl, control; MeS, metabolic syndrome.

A total of 121 participants were included in the study: 61 subjects without malignancy (NM) and 60 subjects with cancer. Subjects comprising NM were stratified into control subjects (Ctrl; *n* = 40) and subjects with metabolic syndrome (MeS; *n* = 21). An overview of the stratification of the study population is shown in Figure 1, and the metabolic profiles are summarized in Table 1.

**Table 1:** Study participants' characteristics.

	**NM (*n* = 61)**	**Ctrl (*n* = 40)**	**MeS (*n* = 21)**	**pCC (*n* = 22)**	**CC (*n* = 38)**
	**Median (95% CI)**				
Age (years)	54 (50–57)^a, b^	53 (47–57)^c, d^	55 (51–60)^e, f^	61 (57–72)^a, c, e^	66 (61–68)^b, d, f^
CRP (mg/dL)	0.1 (0.1–0.1)^g, h^	0.1 (0.1–0.1)^i, j^	0.2 (0.1–0.3)^k^	0.6 (0.4–1.8)^g, i^	3.3 (1.0–5.4)^h, j, k^
Albumin (g/dL)	44.7 (43.1–46.3)^l, m^	44.7 (42.8–47)^n, o^	44.3 (41.7–47.3)^p, q^	38.9 (35.6–41.6)^l, n, p^	34.4 (31.3–36.3)^m, o, q^
Hemoglobin (g/dL)	15.3 (14.9–15.6)^r^	15.3 (14.9–15.6)^t^	15.5 (14.4–15.7)^u^	14.6 (14.1–15.6)^s, v^	13.1 (12.3–13.5)^r, s, t, u, v^
Waist circumference (cm)	101 (97–104)^w^	97 (94–101)w	106 (101–113)	103 (95–111)	101 (96–106)
Blood pressure—systolic (mmHg)	135 (130–140)	133 (125–139)	142 (130–156)	140 (134–154)^x^	130 (120–144)^x^
Blood pressure—diastolic (mmHg)	86 (82–90)^y^	85 (80–88)	92 (85–95)^&^	89 (80–95)^z, §^	80 (75–86)^y, z, &, §^

*Adjusted P-values: <0.0001: b, d, g, h, i, j, m, o, q, r, t, u; <0.005: a, c, l, n, w, &; <0.05: e, f, p, s, v, x, y, z, §*.

Cancer patients were assigned to either pCC (*n* = 22) or CC (*n* = 38) according to the extent of body weight loss. All subjects in CC reported body weight loss of ≥ 5% during the last 6 months. The numbers of patients with C-reactive protein (CRP) ≥ 0.5 mg/dL, plasma albumin <3.2 g/dL, or hemoglobin <12 g/dL are summarized in Table 2. Subjects belonging to the Ctrl and MeS groups within NM were about the same age, as were cancer patients belonging to pCC or CC. Subjects of NM were significantly younger than cancer patients of either pCC or CC (*P* = 0.0002; *P* < 0.0001, respectively). Results represent *P*-values corrected for age as a confounding variable.

**Table 2:** Characteristics of cancer patients meeting the criteria CRP ≥ 0.5 mg/dL; plasma albumin <3.2 g/dL; hemoglobin <12 g/dL; or none of the criteria.

	**pCC (*n* = 22)**	**CC (*n* = 38)**
	***n*** **(%)**	***n*** **(%)**
CRP ≥ 0.5 mg/dL	14 (63.5)	32 (84.1)
Plasma albumin <3.2 g/dL	1 (4.5)	14 (36.8)
Hemoglobin <12 g/dL	2 (9.0)	7 (18.4)
None	8 (36.4)	5 (13.2)

Table 3 shows the types of cancer entities and stages diagnosed in subjects comprising pCC and CC. In this study, 90.0% of pCC and 65.8% of CC patients suffered from lung cancer. Moreover, 40.9% of pCC and 65.8% of CC patients had been diagnosed with stage IV malignancies. Since classification was not part of the recruitment process^6^, subjects newly diagnosed with cancer were classified as pCC and CC a posteriori. Notably, almost two-thirds of subjects (63.3%) were already cachectic at the time of cancer diagnosis.

**Table 3:** Cancer entities and stages.

	**pCC (*n* = 22)**	**CC (*n* = 38)**
**Cancer entities**	***n*** **(%)**	***n*** **(%)**
Lung cancer	20 (90.0)	25 (65.8)
Pancreatic cancer	1 (4.5)	3 (7.9)
Other malignancies of the GI tract	1 (4.5)	7 (18.4)
Lymphoma	0 (0.0)	3 (7.8)
**Cancer stage**		
I	2 (9.1)	1 (2.6)
II	5 (22.7)	4 (10.5)
III	6 (27.3)	4 (10.5)
IV	9 (40.9)	25 (65.8)
n.d.	0 (0.0)	4 (10.5)

The proportion of subjects on antidiabetics (metformin, glimepirid) and lipid-lowering drugs (statins) at the time of their enrollment was higher for both pCC and CC compared to NM and higher in CC than pCC, with 44.7% of cancer patients in CC on antidiabetic drugs vs. 9.1% in pCC, and 39.4% of CC on lipid-lowering drugs vs. 22.7% of pCC (Table 4). In the NM group, one subject was taking lipid-lowering medication and one subject was taking antidiabetic medication.

**Table 4:** Medications.

	**NM (*n* = 61)**	**Ctrl (*n* = 40)**	**MeS (*n* = 21)**	**pCC (*n* = 22)**	**CC (*n* = 38)**
	***n*** **(%)**				
Antidiabetic drugs	1 (1.6)	0 (0.0)	1 (4.8)	2 (9.1)	17 (44.7)
Lipid-lowering drugs	1 (1.6)	0 (0.0)	1 (4.8)	5 (22.7)	15 (39.4)

### Analysis of Group-Specific Differences

We first analyzed whether NM, pCC, and CC differed in their plasma levels of Glc, TG, HDL-C, LDL-C, and Chol (corrected for age as a confounder) (**Figure 3**). In the ensuing paragraphs, the results of each of the parameters are shown for:

- Three-group analysis (NM, pCC, and CC);
- Four-group analysis (NM stratified into the subgroups Ctrl and MeS), which explored whether the results from the three-group analyses hold true for both Ctrl and MeS or have to be restricted to one of these subgroups;
- Effect of antidiabetic or lipid-lowering medications on results of the first two analyses.

#### Fasting Plasma Glucose

##### Three-group analysis (3GA)

**Figure 2:**
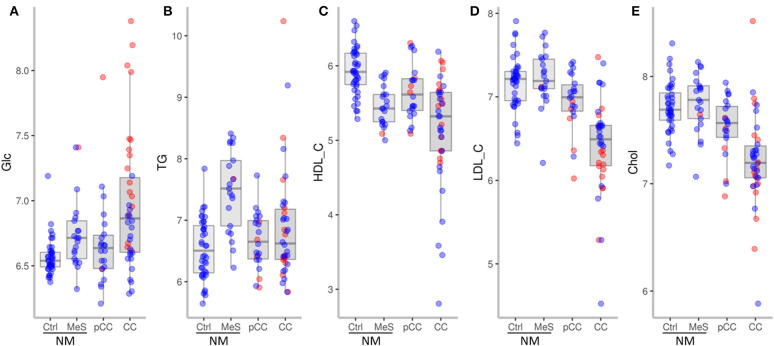
Plasma levels (log_2_ mg/dL) of glucose **(A)**, triglycerides **(B)**, HDL-cholesterol **(C)**, LDL-cholesterol **(D)**, total cholesterol **(E)** in NM, Ctrl, MeS, pCC, and CC. Red dots represent subjects on antidiabetic medication **(A)** or on lipid-lowering medication **(B–E)** at the time of enrollment.

Glc was significantly increased in CC compared to NM (adj. *P* = 0.015) and pCC (adj. *P* = 0.010) (Figure 2A).

##### Four-group analysis (4GA)

Stratifying NM subjects according to the presence of metabolic syndrome revealed that only Ctrl was significantly different from CC (adj. *P* = 0.007) and pCC (adj. *P* = 0.017), but that MeS was not (adj. *P* = 0.516 and 0.578, respectively). Nevertheless, though fasting glucose level of ≥ 100 mg/dL was a secondary criterion for assignment to MeS, there was no significant difference between MeS and Ctrl (adj. *P* = 0.347). Thus, upon stratification, the result obtained in 3GA holds true only for the Ctrl subfraction.

##### Effect of antidiabetic or lipid-lowering medications (Meds)

**Figure 3:**
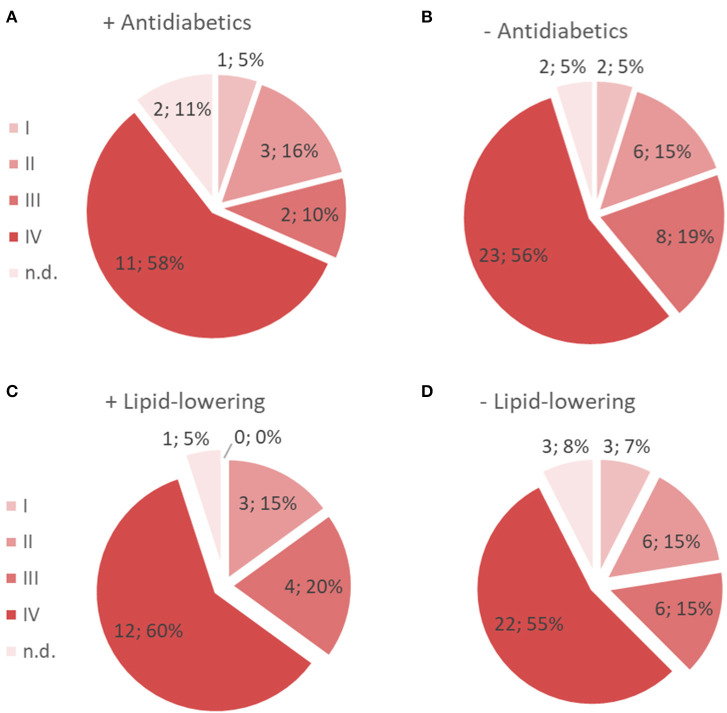
Prevalence of cancers of different stages of patients who were on antidiabetics **(A)** and those who were not **(B)**, as well as of patients who were on lipid-lowering medication **(C)** and those who were not **(D)**.

As shown in Table 4, the proportion of subjects taking antidiabetic medication varied markedly between groups. In CC, 44.7% (*n* = 17) of subjects were on antidiabetics as compared to 9.1% (*n* = 2) in pCC; 4.8% (*n* = 1) in MeS; and none (*n* = 0) in Ctrl. In order to estimate the effect of antidiabetic medication on Glc, we included the medication as a second putative confounding variable (in addition to age) in our multiple regression models. Interestingly, neither the three-group nor the four-group comparisons yielded any significant differences between groups, suggesting that the increase in glucose in CC was due to cancer patients on antidiabetic medication. Notably, 58% of the cancer patients on antidiabetic medication, vs. 56% not taking antidiabetics, were classified as stage IV. These almost equal percentages indicate that intake of antidiabetics did not correlate with tumor stage, but rather with the severity of cachexia (Figures 3A,B).

#### Triglycerides

##### 3GA

Once corrected for age as a confounder, TG values were not significantly different between NM, pCC, and CC (Figure 2B).

##### 4GA

Stratifying NM subjects into Ctrl and MeS revealed that triglyceride levels were significantly lower in pCC (adj. *P* = 0.0047) and CC (adj. *P* = 0.0436) compared to MeS. A level of plasma triglycerides of ≥ 150 mg/dL was a criterion for stratification into MeS applied in this study. As might be expected, TG values also were significantly increased in MeS compared to Ctrl (adj. *P* = 0.0416).

##### Meds

A significant proportion of cancer patients, particularly in CC (39.4%) and, to a lesser extent, in pCC (22.7%), were on lipid-lowering drugs. Lipid-lowering medication had little effect on triglyceride levels (*P* = 0.934). Including the medication as a confounder in the models decreased the significance levels a little, likely due to the increasing degrees of freedom, and thus, numbers of statistical tests for which to correct (adj. *P*-values of 0.056 and 0.072 for the comparisons CC vs. MeS and vs. Ctrl, respectively, are no longer significant). Notably, 60% of cancer patients on lipid-lowering medication, vs. 55% who were not, had been classified as stage IV, only a slight increase in the former group over the latter (Figures 3C,D).

#### HDL-C

##### 3GA

HDL-C was significantly decreased in CC compared to NM (adj. *P* < 0.001) and pCC (adj. *P* = 0.001) (Figure 2C).

##### 4GA

Stratifying subjects of NM into Ctrl and MeS revealed that HDL-C was significantly lower in CC compared to Ctrl (adj. *P* < 0.001) and pCC (adj. *P* = 0.001), but not compared to MeS. An HDL-C level of <40 mg/dL was a criterion for stratification into MeS applied in this study and HDL-C was significantly decreased in MeS compared to Ctrl (adj. *P* = 0.004), which accounts for this result.

##### Meds

Including lipid-lowering medication in the models as a confounder had no effect on significance levels of HDL-C between groups as reported above, although HDL-C was significantly lower in CC also compared to MeS (adj. *P* = 0.0352).

#### LDL-C

##### 3GA

LDL-C was significantly decreased in CC compared to NM and pCC (adj. *P* < 0.001 for both) and tended to be lower in pCC compared to NM (adj. *P* = 0. 054) (Figure 2D).

##### 4GA

LDL-C did not differ between Ctrl and MeS, thus, four-group analysis yielded analogous results, whereby LDL-C was significantly lower in CC compared to Ctrl, MeS, and pCC (adj. *P* < 0.001 for all).

##### Meds

Though lipid-lowering drugs significantly reduced LDL-C (*P* = 0.025), when lipid-lowering medication was included in the models as a confounder, we obtained results similar to HDL-C.

#### Total Cholesterol

##### 3GA

Chol was significantly decreased in CC compared to NM and pCC (adj. *P* < 0.001 and adj. *P* = 0.001, respectively) (Figure 2E).

##### 4GA

Chol did not differ between Ctrl and MeS; thus, four-group analysis yielded analogous results, whereby total cholesterol was significantly lower in CC compared to Ctrl and MeS (*P* < 0.001 for both), and also pCC (adj. *P* = 0.003).

##### Meds

Lipid-lowering drugs did not significantly affect cholesterol levels over all patients, therefore including them as a confounder to the models did not affect results comparing different groups.

## Discussion

Cancer cachexia is associated with extensive metabolic disturbances (9). Insulin resistance in cancer patients is demonstrated by decreased insulin sensitivity or impaired glucose tolerance (34) and is suspected to increase during cachexia progression (35, 36). Moreover, elevated glucagon levels (37) promote hepatic gluconeogenesis, thereby increasing plasma glucose levels in cancer cachexia (37–39). However, others state that glucose levels are unaltered in cancer cachexia (40, 41) possibly reflecting a complex metabolic dynamic in cancer cachexia (42).

Here, we explored fasting plasma glucose levels, whose impairment is the second sign of insulin resistance (independent of impaired glucose tolerance) (43). Supporting the notion of increased insulin resistance in cancer cachexia, our findings showed levels of plasma fasting glucose significantly increased in cachectic cancer patients. Intriguingly, however, while we obtained similar Glc results when we stratified subjects without malignancy into NM with and without MeS, we found that the increase in plasma glucose in cancer cachexia was due to subjects on antidiabetic medication. When we excluded cancer patients on antidiabetic medications from our analysis, the difference between groups vanished. This discrepancy in results reflects the pronouncedly higher proportion of CC patients on antidiabetic medication compared to pCC patients and subjects without malignancy. Our results suggest that the increase in Glc is associated with cachexia progression rather than cancer stage. High levels of fasting glucose at the time of cancer diagnosis have been associated with poor prognosis for patients with non-small-cell lung carcinoma (NSCLC) (44), which supports the notion that an elevated level of fasting plasma glucose is not only a measure of metabolic disturbance or insulin resistance, but a marker of poor prognosis.

Cancer cachexia mechanisms promote lipid mobilization (9). Lipid-lowering drugs affected LDL-C but not TG, HDL-C, and Chol. The proportion of cachectic cancer patients on lipid-lowering drugs was significant, with almost twice as many CC as pCC on hypolipidemic medications. Analogous to our findings for glucose, this might indicate that lipid-lowering drugs are being prescribed, at least in part, due to the effects of cachexia, while cancer remains subclinical.

We found no differences in TG between subjects without malignancy and pre-cachectic and cachectic cancer patients, which is in accordance with findings on lung cancer patients (26). Stratifying the subjects without malignancy into two distinct subgroups confirmed that subjects with metabolic syndrome had significantly higher triglycerides than those without MeS. Taking MeS into consideration revealed a slight but significant shift in TG levels toward NM subjects in pCC, but not CC, patients. This might signify a reduction of triglycerides in pre-cachexia, presumably due to a lipid-demanding mechanism, which would remain unnoticed unless stratifying subjects without malignancy into Ctrl and MeS groups.

In contrast to triglycerides, Chol was significantly reduced in cachectic cancer patients, which aligns with previous findings (25, 26). HDL-C and LDL-C also were reduced. The decrease of Chol, HDL-C, and LDL-C in CC compared to pCC patients might be caused by a pathophysiological mechanism, which preferentially and swiftly removes Chol from the systemic circulation, particularly compared to TG. The steepest decrease occurred in Chol and LDL-C, induced mainly by lipid-lowering drugs for LDL-C. In the NM group, HDL-C was significantly lower in MeS subjects than in Ctrl. Stratification of NM revealed a key difference: of Chol, HDL-C, and LDL-C; the two parameters Chol and LDL-C were robust and independent of MeS prevalence in the NM cohort, while CC became similar to MeS with regard to HDL-C. If cachexia did not influence HDL-C values in CC, this group would be expected to be similar to NM, not MeS. Thus, the significant decrease of HDL-C in cachectic cancer patients was only due to subjects without metabolic syndrome. Notably, though not statistically significant, there was a clear tendency for Chol and LDL-C to decrease in pCC compared to the non-malignant state. Thus, if a patient's medical history shows normal or elevated Chol and LDL-C, an inexplicable decrease in both parameters (even if values remain higher than normal) might indicate subclinical malignancy. This hypothesis requires further investigation. However, it has indeed already been noted that cholesterol levels begin to decline years prior to cancer diagnosis (25).

## Conclusions

Starting with pathomechanisms of cachexia, we hypothesized that metabolic plasma parameters might differ between the non-malignant state and pre-cachexia/cachexia in cancer.

Glc increases were seen only in cachectic cancer patients on antidiabetics. Whether this observation is of clinical relevance or prognostic value requires further research. In summary, our study supports the notion that metabolic plasma parameters reflecting metabolic dysregulation associated with cachexia progression might be useful markers for early detection of malignancy.

We found that LDL cholesterol and total cholesterol might indicate cachexia stages irrespective of the presence of metabolic syndrome- in contrast to triglycerides and HDL cholesterol- in non-malignant subjects and lipid-lowering medication, which is particularly frequent in cancer patients. Though not statistically significant, the decrease in the values of these parameters manifests in pre-cachexia; if this decrease proves to be reproducible and statistically significant, LDL-C and Chol might prove to be useful for early detection of malignancy. This would require longitudinal studies of these parameters across larger cohorts and longer periods of time to investigate whether, for example, a drop in Chol and LDL-C compared to earlier values in the same subject, which cannot be ascribed to other causes such as a change in lifestyle or starting LDL-C-lowering medication, might indicate early malignancy.

## Data Availability Statement

The datasets generated for this study are available on request to the corresponding author.

## Ethics Statement

The studies involving human participants were reviewed and approved by Ethics Committee of the Federal State Lower Austria (GS1-EK-4/290-2014). The patients/participants provided their written informed consent to participate in this study.

## Author Contributions

HZ: conceptualization. HZ and EZ-T: data curation. HZ and DP: formal analysis. EZ-T and MP: funding acquisition. EZ-T: project administration. HZ and DP: visualization. MP: supervision. HZ: writing—original draft. KH, HK, E-CK, BM, DP, HS, TS, EZ-T, and MP: writing—review & editing. All authors contributed to the article and approved the submitted version.

## Conflict of Interest

The authors declare that the research was conducted in the absence of any commercial or financial relationships that could be construed as a potential conflict of interest.

## Footnotes

^1^Since it is not known whether all pre-cachectic cancer patients will develop cachexia, we use the term pre-cachexia herein to include non-cachexia.

^2^Ultimately our efforts to recruit subjects of similar ages across cohorts were not successful (see Section Description of Study Population), so we applied a statistical correction for age in Approach 1.

^3^Glucose concentration was enzymatically measured using the hexokinase reaction via ATP consumption.

^4^Triglycerides, total cholesterol, HDL and LDL cholesterol, C-reactive protein (CRP), albumin, and hemoglobin were measured using standard laboratory diagnostic methods.

^5^The multcomp package allows for multiple comparisons of k groups in general linear models (http://math.furman.edu/~dcs/courses/math47/R/library/multcomp/html/multcomp.html).

^6^For details, see Section Materials and Methods.
